# Ribonucleic acid (RNA) biosynthesis in human cancer

**DOI:** 10.1186/s12935-015-0167-3

**Published:** 2015-02-21

**Authors:** Omar S Hajjawi

**Affiliations:** Department of Biology, Arab American University, P. O. Box 240, Jenin, Israeli Occupied Territories of Palestine

**Keywords:** Polymerases, Polyadenylation, Ribosomal RNA, Telomerase RNA, Aptamers, Carcinogenesis, Genometastasis, Antineoplastic agents, Signal Transducer and Activator of Transcription-STAT, Drug resistance

## Abstract

In many respects, the most remarkable chemical substances within the genome of eukaryotic cells are remarkable proteins which are the critical structural and functional units of living cells. The specifications for everything that goes in the cell are natural digital-to-digital decoding process in an archive sequence by deoxyribonucleic acid (DNA) and an articulate construction by ribonucleic acid (RNA). The products of DNA transcription are long polymers of ribonucleotides rather than deoxyribonucleotides and are termed ribonucleic acids. Certain deoxyribonucleotide sequences, or genes, give rise to transfer RNA (tRNA) and other ribosomal RNA (rRNA) when transcribed. The ribonucleotide sequences fold extensively and rRNA is associated with specific proteins to yield the essential cell components, ribosomes. Transcription of other special sequences yields messenger RNAs (mRNAs) that contain ribonucleotide sequences that will be ultimately translated into new types of amino acid sequences of functional cellular protein molecules. This switch to a different variety of cellular molecular sequences is complex, but each sequence of the three ribonucleotides specifies the insertion of one particular amino acid into the polypeptide chain under production. Whilst mRNA is considered the vehicle by which genetic information is transmitted from the genome and allocated in the appropriate cytoplasmic sites for translation into protein via cap-dependent mechanism, the actual translation depends also on the presence of other so-called household and luxury protein molecules. Recent evidence suggests RNA species are required at initiation, because treatment of cells with antibiotics or drugs that inhibit RNA synthesis cause a decrease in protein synthesis. The rRNA is necessary as a structural constituent of the ribosomes upon which translation takes place, whereas tRNA is necessary as an adaptor in amino acid activation and elongation protein chains to ribosomes. In this article, we review malignant tumor, with stem like properties, and recent technical advances into the phenomenon of micro-particles and micro-vesicles containing cell-free nucleic acids that circulate plasma. New areas of research have been opened into screening tumor telomerase progression, prognosis of aptamers targeting cell surface, monitoring the efficacy of anticancer therapies, oncogenic transformation of host cell, and RNA polymerases role in the cell cycle progression and differentiation.

## Introduction

**Figure 1:**
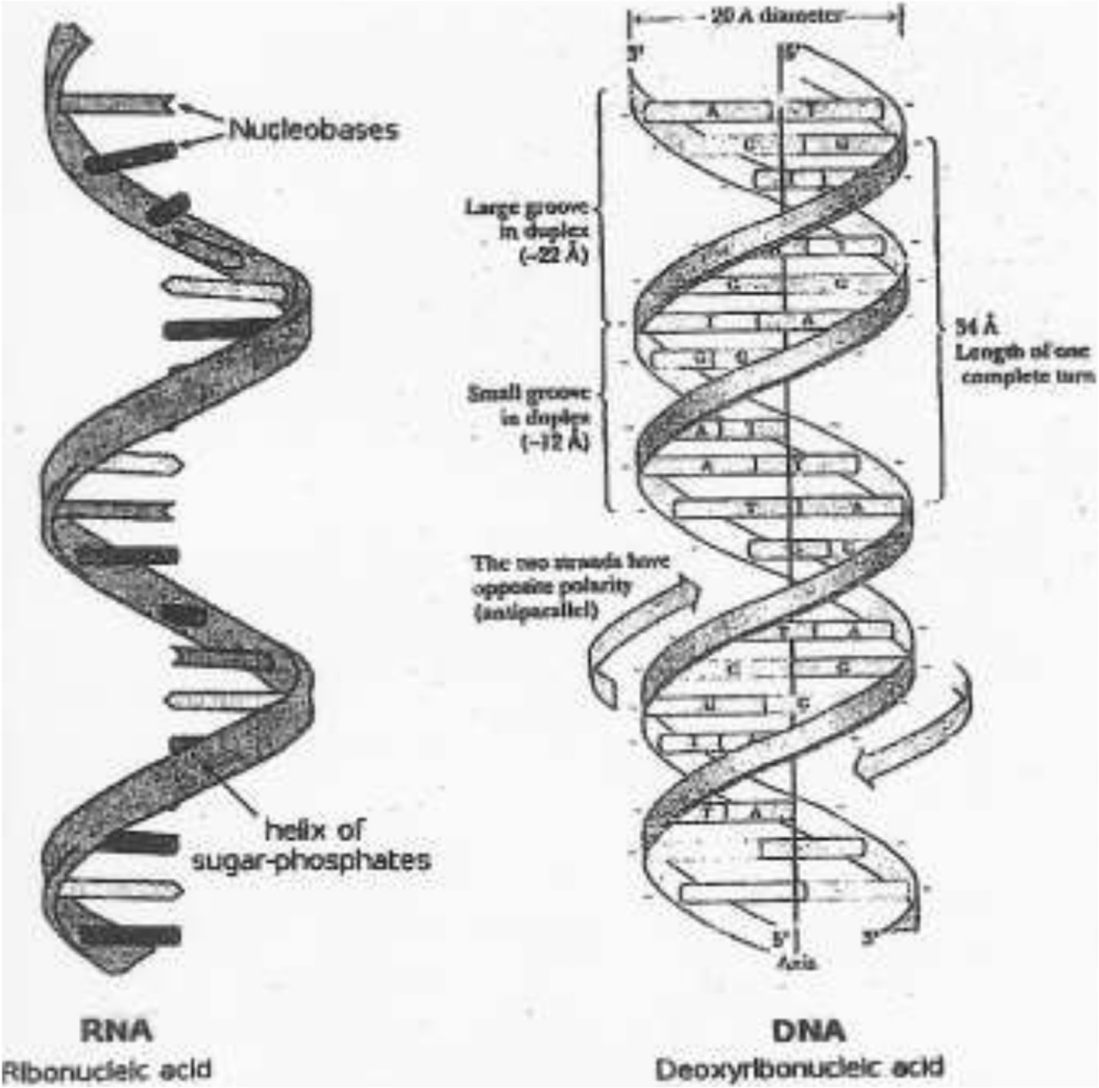
**The essential single strand RNA and two strands DNA nucleic acids.**

Rudolf Virchow (1821–1902) is generally credited as the first to recognize leukemia cells [1], and Fridrich Miescher (1844–1895) had identified and isolated cellular substance containing nitrogen and phosphorus, whereas Albrecht Kossel (1853–1927) isolated the nucleic acids: two purines (adenine and guanine) and three pyrimidines (thymine, cytosine and uracil) [2]. Cancer is however defined as a group of more than 100 different diseases that is caused by multiple changes in cellular DNA and RNA, and it is characterized by uncontrollable growth (mitosis) in which cells are aggressive, invasive and sometimes metastatic [3]. Also, the discovery of the double helix of DNA molecule by Watson and Crick [4] was an added milestone in the twentieth century science (Figure 1). The genetic material of living organisms and double helix of DNA molecule formed the foundation of new discipline of molecular biology.

A complete turn of DNA double helix spans ten base pairs that cover a distance of 34 Ǻ (3.4 nm). The individual base pairs are spaced 34 Ǻ (3.4 nm) apart. The places where the strands cross hide base pairs that extend perpendicular to the viewer. The inside diameter is 11 Ǻ (1.1 nm), and the outside diameter 20 Ǻ (2.0 nm). Within the cylindrical outline of the double helix are two grooves that are large enough to house polypeptide chains. The largest human chromosome, chromosome number 1, consists of approximately 220 million base pairs and is 85 nm long. The minus signs alongside the double helix strands represent many negatively charged phosphate groups along the entire length of each strand. Unlike double-stranded DNA, RNA is a single-stranded molecule in many of its biological roles and has a much shorter chain of nucleotides. However, RNA can, by complementary base pairing, form intra-strand double helixes, as in tRNA. While DNA contains deoxyribose, RNA contains ribose (in deoxyribose there is no hydroxyl group attached to the pentose ring in the 2′ position). These hydroxyl groups make RNA less stable than DNA because it is more prone to hydrolysis. The complementary base to adenine is not thymine, as it is in DNA, but rather uracil, which is a nunmethylated form of thymine.

*Adopted from:* Watson J.D. and Crick, F.H.C. (1953) “Molecular structure of nucleic acid. A structure of deoxyribosenucleic acid”, Nature, vol.171, pp.737-738; Gregory, S., Barlow, K.F., McLay, K.E., Kaul, R., Swarbreck, D., Dunham, A., Scott, C.E., Howe, K.L. and Woodfine, K. (2006). “The DNA sequence and biological annotation of human chromosome 1”. Nature, vol. 441 (7091), pp.315–221; Nelson, D.L. and Cox, M.M. (2008) Lehninger Principles of Biochemistry, 5th edn, pp.277-287. New York, NY: W.H. Freeman and Company.

The detailed mechanism by which such genetic material could be expressed as the structural and catalytic proteins which play so important a role in the functioning of all living cells was still not obvious [5-7]. Stanley miller (1930–2007) and Harold Urey (1893–1981) designed an experiment that simulated hypothetical conditions thought to be present at the time of early life on earth [8,9] and tested qualitatively for the occurrence of earthly chemicals that originated life [10]. The essential chemical elements of H_2_, CH_4_, H_2_O and NH_3_ which were confined to a sterile glass flasks and tubes, were subjected to electric discharges and the interaction produced 20 amino acids, the building block of proteins, as well as other organic compounds like: adenosine triphosphate, lipids, some sugars and the bases for RNA and DNA (Lazcano and Bada [11,12]).

Crick et al. [13] designed an elegant experimental strategy to determine the nature of the genetic code that was remarkably the correct one despite the absence of technology to analyze and compare DNA and protein sequence. The genetic code is the relation between the bases sequence in DNA (or its RNA transcripts) and amino acids sequence in proteins [14]. The features of the genetic code are as follows: (1) three nucleotide encode an amino acid, (2) the code is nonoverlapping, (3) the code has no punctuation, and (4) the genetic code is degenerate [14-16].

Proline only differs from this basic structure as it contains an unusual ring to the N-end amine group, which forces the CO–NH amide moiety into a fixed conformation [17,18]. Once this conceptual breakthrough had been made, the complex task of unraveling the many steps in protein biosynthesis could begin in the laboratory [19]. The sequence of amino acids in a protein is defined by a gene and encoded in the genetic code. Although this genetic code specifies 20 different L-α-amino acids, the residues in a protein are often chemically altered in post-translational modification: either before the protein can function in the cell, or as part of control mechanisms [17,20].

Genes are made of nucleic acids that contain the instructions for making proteins; enzymes are also made of proteins and they are needed to replicate genes [21-25].

Genetic information encoded in the two complementary strands of the DNA of any structural gene is transcribed by an enzyme called DNA-dependent RNA polymerase that catalyzes the synthesis of RNA from a DNA or RNA template [26]. The eukaryotic RNA polymerases (pol-I [27], pol-II [28] and pol-III [29,30] are the central multiprotein machines. The DNA-dependent RNA polymerase makes a single stranded RNA copy, complementary to one of the strands that are called mRNA. This attaches to a subcellular organelle ribosome which is composed of two subunits between 25 and 30 nm (250–300 Å) in diameter with an rRNA to rotein ratio that is close to 1 [18,19]. It operates as a black box upon which the mRNA is translated [31,32]. The term translation encompasses all the steps by which the genetic content of the mRNA contained in the linear sequence of ribonucleotides is converted into a linear sequence of amino acids [26]. Whilst mRNA might be considered the means whereby genetic information is actually transmitted from the genome (the DNA), and placed in the appropriate cytoplasmic sites for translation into protein [33]. Organelle biogenesis and maintenance requires newly synthesized proteins, each of which needs to go from the ribosome translating its mRNA to the correct translocation to an organelle sub compartment [34]. Interestingly, it was demonstrated that fat and obesity-associated gene is located on chromosome 16 of mRNA demethylase [E.C. 2.1.1.270], i.e. methylation of mRNA plays a critical role in human energy homeostasis [35-37].

The nucleic acids are assembled from individual nucleotides just as proteins are assembled from individual amino acids. The nucleotides are synthesized by a series of enzyme mediated-mediated reactions [26,38,39]. Biochemical pathways are separately pursued for the synthesis of the ribose and the different bases that are then assembled to form nucleotide triphosphates [40]. The energy-carrying molecule ATP which consists of the base adenine, ribose, and three phosphate groups is also one of the nucleotide building blocks used in the synthesis of RNA, and the others being guanosine triphosphate, cytidine triphosphate and uridine triphosphate [41]. These nucleotides are usually synthesized by the transfer of energy from ATP to their diphosphate forms of the nucleotides [18]. Thus, there is a general pool of nucleotide triphosphates that work as building blocks for RNA within the cell [42,43]. These free nucleotides assembled into a linear sequence where the RNA molecule contains some of the coded information that is present in DNA, and when the DNA is copied into mRNA using base pairing of adenine-thymine, and guanine-cytosine is the process of DNA transcription [14]. During the synthesis of mRNA, the bonds between these pairs in DNA, adenine-thymine and guanine-cytosine, and the double- stranded structure partially unwinds and the two strands separate [18]. The bases of the free nucleotide triphosphates and the bases in one of the separated DNA chains form new bonds. DNA therefore acts as a template to command the sequence of the bases in RNA. The base adenine in the free nucleotide would pair with the base thymine in DNA, and the base uracil in the free nucleotide would also pair with the base adenine in DNA. Similarly, the base cytosine in the free nucleotide pairs with the base guanine in DNA, and the base guanine in the free nucleotide pairs with base cytosine in DNA [44]. The resulting outcome would be a new sequence of bases in RNA which is an enantiomer mirror image of the base sequence in DNA [43]. Since the primary advantage of nucleotide base-pairing is that DNA two strands can replicate easily and accurately, each base can only pair to one other base (thymine to adenine, adenine to thymine, cytosine to guanine, and guanine to cytosine). Thus, if the original DNA codon contains the base sequence cytosine-guanine-thymine, the complementary codon sequence in mRNA is guanine-cytosine-adenine [45-48].

Once the appropriate free nucleotide triphosphates are base-paired to the corresponding bases in DNA, the nucleotides are joined to each other by the enzyme RNA-polymerase II (12 subunits) that causes pyrophosphate to be split off from nucleotide triphosphate in the process of linking one nucleotide to the next, forming the sugar-phosphate backbone of mRNA [43,49,50]. This enzyme is active only in the presence of DNA and it does not link the free nucleotide triphosphates together in its absence [46]. The enzyme moves along the DNA strand, linking one nucleotide at a time into the growing mRNA chain [47,51]. RNA-polymerase II activity is DNA-dependent, meaning that it must have a DNA template molecule before it can synthesize the RNA transcript. The DNA-dependent polymerase must also have Mg^2+^ and ribonucleoside 5′ triphosphates in order to carry out RNA synthesis. The RNA polymerase creates the new RNA strand from 5′ to 3′ [48].

Protein expression is determined by the rate of transcription and by post transcription processes that lead to changes in the mRNA transport, stability and translation efficiency [52]. These post-transcriptional processes are mediated by RNA modifications, secondary structure, micro RNAs (miRNAs), and RNA-binding proteins that recognize regulatory elements located in the 3′ untranslated regions of transcripts [53]. The critical cellular process of polyadenylation that is the addition of poly (A) tail to mRNA that plays important roles in many aspects of the cellular metabolism of mRNA, though it begins as the transcription of a gene finishes or terminates. The 3′-most segment of the newly made pre-MRNA is first cleaved off by a set of proteins; these proteins then synthesize the poly (A) tail at any one of several possible sites [54]. The cleavage generates the free 3′-hydroxyl group that defines the end of the mRNA to which adenine residues are immediately added by polyadenylate polymerase that catalyzes the reaction:

$$ \mathrm{R}\mathrm{N}\mathrm{A} + \mathrm{nATP} \circ ledR\ \mathrm{R}\mathrm{N}\mathrm{A}\hbox{-} {\left(\mathrm{A}\mathrm{M}\mathrm{P}\right)}_{\mathrm{n}} + {\mathrm{n}\mathrm{P}\mathrm{P}}_{\mathrm{i}} $$

where n = 200–250 [18,55]. The poly (A) tail and its associated proteins are more likely to ptotect mRNA from enzymic destruction [56]. Protein-coding genes may have more than one polyadenylation site, “extra RNA”, so a gene can code for several mRNAs that differ in their 3′-end [57], though mRNA polyadenylation is controlled by various *cis*-acting elements surrounding the cleavage site and their binding factors. Since alternative polyadenylation changes the length of the 3′ untranslated region, global shortening of 3′ untranslated regions through alternative polyadenylation is an emerging hallmark of cancer [58,59]; it can also change which binding sites for miRNAs the 3′ untranslated region contains [60]. So, polyadenylation is a way of marking the RNA for degradation for many non-coding RNAs, including tRNA, rRNA,snRNA and snoRNA [61]. The RNA exosome (30-100 nm) is a conserved degradation machinery, which obtains full activity only when associated with cofactors; poly (A) tails have been found on human RNA fragments of both homopolymeric and mostly heterpolymeric tails [61-63]. Regulated polyadenylation of specific mRNAs is involved in oogenesis, cell cycle progression and synaptic plasticity [64]. Many polyadenylation *trans*-acting factors, including polyadenylate polymerase, are increasingly found to be involved in cell cycle, apoptosis and cancer prognosis [65]. So, genes undergoing alternative cleavage and polyadenylation during human cancer progression may be useful novel biomarkers and potentially targeted for disease prevention and treatment [66,67].

The micro RNAs are an endogenous class of post-transcriptional regulators that regulate as many as one-third of human genes; they are small in length (21–25 nucleotide-long fragments) and single stranded [68]. Studies suggest that approximately half of known microRNA reside in non-protein coding RNAs (intron and extron) or within the intron of protein coding genes [68,69]. They can recognize and bind to imperfect base-pairing complementary sequences in the 3′-untranslated region of multiple target mRNAs, blocking translation of the gene expression or inducing cleavage of mRNA to control a multitude of critical processes through either reduction or inhibition in the translational efficiency of the target mRNA [70,71]. Recent studies have shown that miRNAs are aberrantly expressed in various human diseases, ranging from cancer to cardiovascular hypertrophy [72]. The micro RNAs target ~60% of all genes, and they are abundantly present to repress 100 s of targets in all human cells; bioinformatics indicates that a 22 nucleotide single stranded RNA composed of 4 different ribonucleotides, they can have over 10^13^ possible sequence combinations. So, since the cell contains typically ~1049 miRNAs, there must be a very high developmental and evolutionary selection pressure that utilizes only specific miRNA oligonucleotide sequences to yield biologically useful miRNA-mRNA interactions [73]. The biogenesis of miRNA is similar to other RNA starting from DNA transcription. A primary miRNA is an independent transcript processed by RNA polymerase II and they are bound in the nucleus by the ‘microprocessor’ complex that consists of ribonuclease III (an Mg^2+^-dependent endonuclease), Drosha, and its co-factor, Pasha (DGCR8) [74]. The generation of mature miRNAs from precursor miRNAs by the ribonuclease III (Dicer1 /TRBP complex in the cytoplasm [75]. Dicer is a specialized ribonuclease that initiates RNA interference by cleaving double-stranded RNA into miRNA fragments [76], and TRBP (the human immunodeficiency virus transactivating response to double strand RNA-binding protein) is an integral component of a Dicer-containing complex [75,76].

Neoplasia that includes many diseases is an abnormality of cellular differentiation, maturation and control of growth [77]. Rupert Allan Willis (1898–1980) defined neoplasm as “an abnormal mass of tissue, the growth of which exceeds and is uncoordinated with that of the surrounding normal tissues and persists in the same excessive manner after cessation of the stimuli that evoked the change”, and this definition is the widely cited one [29]. Also, several neoplastic and non-neoplastic diseases were shown to contain circulating nucleic acids and that in cancer they originate mostly from tumor [78]. Hence, the level of circulating nucleic acids that have been associated with tumor burden and malignant progression, are utilized for cancer screening, prognosis, and monitoring the efficacy of an anticancer therapy [79].

Also, Conrad H. Waddington (1905–1975) had reported an intricate interplay between the cellular environment and genes effects on phenotype determination; he attributed the molecular signals to epigenetic phenomenon [80-82]. The epigenetic signals that are responsible for the establishment, maintenance and the reversal of metastable transcriptional states, have direct correlation with promoter hypermethylation and silenced tumor suppressor genes, upstream transcription factors and DNA repair enzymes [83,84]. Since cancer is ultimately a disease of genes, the mechanism by which epigenetic information is transmitted through cell division remain unclear as the complex epigenetic states are orchestrated by several converging signals [84-86].

**Figure 2:**
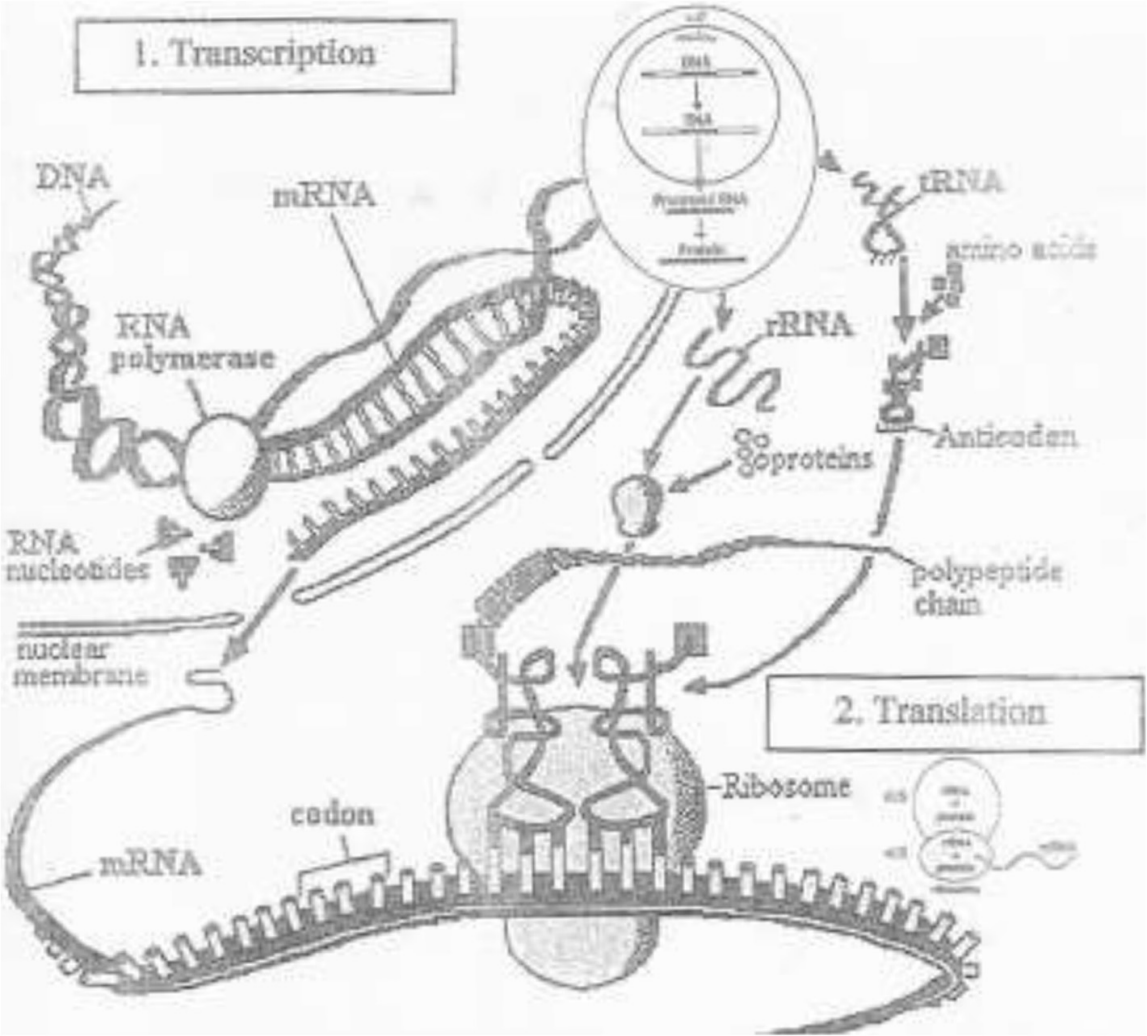
**The fundamentals process of information transfer in cells.**

The biologically active RNAs, including mRNA, tRNA, rRNA, small nucleic RNAs, [87] and other non-coding RNAs [64], contain self-complementary sequences that allow parts of the RNA to fold [88-90] and pair with itself to form double helices (Figure 2).

The analysis of these RNAs has revealed that they are highly structured and they do not consist of long double helices but rather collections of short helices packed together into structures akin to proteins. RNAs fold and conform to enzyme chemical catalysis [88,91,92], for example, the active site of ribosome that analyzes peptide bond formation and release consists entirely of RNA [87,93]. The rRNA is necessary as a structural component of ribosomes upon which translation actually takes place and tRNA is required in amino acid activation, as an adaptor in mRNA directed amino acid specification and in binding the growing protein chains to the ribosomes (Figure 2). In the process of DNA transcription the positioning of nucleotide units in the RNA molecules being made is under the control of the DNA that acts as a template [18,94]. The means by which this template dictates such a sequence involves both base pairing interactions and specific interactions between proteins and nucleic acids [95]. Each RNA chain is initiated at a specific site on the DNA template and subject to termination at another unique type of site on the template, i.e. there are defined units of transcription [41,96]. It is a selective process. Specific signals in the DNA template are recognized by the transcription apparatus. Initiation is governed by promoter regions in the DNA, and the region governing termination is designated a terminator [97].

(1) Transcription: Information encoded in the nucleotide sequence of DNA is transcribed through synthesis of mRNA whose sequence is dictated by the DNA sequence. (2) Translation: As the sequence of mRNA is decoded by the protein synthesis machinery, it is translated into the sequence of amino acids in a protein. This information transfer is encapsulated in the dogma: DNA → RNA → Protein.

*Adopted from*: Hernández, G. (2012) On the Emergence and Evolution of the Eukaryotic Translation Apparatus, in Cell-Free Protein Synthesis, Biyani, M. (ed.), p.32. Retrieved September13, 2014 from http://cdn.intechopen.com/pdfs-wm/39965.pdf.

Archibald Garrod (1857–1936) was one of the first scientists to propose that inherited factors (genes) controlled the function of proteins [51]. Defects (diseases) in metabolism could be linked to the failure of specific enzymes to catalyze essential biochemical reactions. Protein synthesis, translation, is directed by an mRNA molecule. Translation can be seen to occur in two phases: (1) information transfer, in which RNA base sequence of the mRNA determines the sequence of amino acids and (2) chemical processes, in which the peptide bonds between the adjacent amino acids are formed. The components required for translation include: mRNA, ribosomes (60S and 40S), tRNA, aminoacyl tRNA synthetases, and accessory proteins involved in initiation, elongation and termination [18,80]. Elongation can be thought to involve three processes: (1) aligning each aminoacylated tRNA, (2) forming the peptide bond to add the new amino acid to the polypeptide chain, and (3) moving the ribosome along the mRNA by three more bases (one codon). Elongation proceeds until a stop codon is reached. There are three stop codons in the genetic code: UAG, UGA, UAA [98].

It is exceptionally difficult to assess the carcinogenic effects of so many agricultural, industrial, and household chemicals, but a significant hazard is posed by the disposal of various agricultural and industrial wastes that may contaminate drinking water, coastal water and marine life pollution [99-101]. Also, the identification of a chemical carcinogen is problematic because of the long lag between chemical exposure and the development of cancer, unless the effect is dramatic [102,103]. In view of the vast number of chemical substances that people encountered during their lives, Table 1 shows most strongly evidenced carcinogenic chemicals.

**Table 1:** **Major chemical carcinogens in humans**

**No.**	**Chemical carcinogen**
1	Aflatoxin - B1 [CAS:1162-65-8] & Aflatoxin - G1 [CAS:1165-39-5]: A group of toxic polynuclear (benzenoid type) metabolite molds produced chiefly by the fungus *Aspergillus flavus*; they are natural contaminants of a wide range of fruits, vegetables, cereal grains and improperly stored food. The B_1_and G_1_ strains are known carcinogens
2	Aromatic amines [CAS: 8007-70-3]: Benzidine-based and naphthyamine are procarcinogens examples that enter the body through the skin, lungs or intestine. The gradation of potency of the carcinogenic amines depends on their hydrophobicity, and on electronic (reactivity, propensity to be metabolically transformed) and steric properties
	Azo dyes [CAS: 84812-61-3]: Any of a broad series of synthetic dyes that have –N = N- as a chromophore group. They are widely used in the food, pharmaceutical, cosmetic, textile and leather industries.
3	Asbestos [CAS: 1332-21-4]: A group of impure magnesium silicate minerals which occur in fibrous form. The various types serpentine, amphibole, amosite and crocidolite are highly toxic by inhalation of dust particles
4	Chemotherapeutic agents: Cytotoxic chemicals that are selectively destructive to malignant cells and tissues. They are classified into antimetabolites, anti-tumour, anthracyclines, topoisomerase inhibitors, plant alkaloids, and alkyting agents which are considered most important because they can add alkyl groups to the many electronegative groups under conditions which are present in some cells to modify DNA chemically. Examples of antineoplastic drugs:
	Busulfan [CAS: 55-98-1]
	Carboplatin [CAS: 41575-94-4]
	Cisplatin [CAS: 9002-60-2]
	Chlorambucil [CAS: 305-03-3]
	Cyclophosphamide [CAS: 50-18-0]
	Mechlorethamine [CAS: 51-75-2]
	Oxaliplatin [CAS: 61825-94-3]
	Thiotepa [CAS: 52-24-4]
5	Heavy metals
	Arsenic ^32^AS [CAS:7440-38-2]
	Cadmium ^48^Cd [CAS: 7440-43-9]
	Chromium ^24^Cr [CAS:7440-47-3]
	Nickel ^28^Ni [CAS:7440-02-0]
6	Hydrocarbons:
	Soot [CAS: 98615-67-9]: An airborne powder made of amorphous carbon, whereas its gas phase contains polycyclic aromatic hydrocarbons.
	Tobacco [CAS: 8037-19-2]: Cured leaves of the species *Nicotiana tabacum* that contains acids (citric, oxalic, formic), alkaloids (nicotine, anabasine, myosmine), carbohydrates (lignin, pentosans, starch, sucrose), as well as tannin, ammonia, glutamine, and micro amounts of zinc, iodine, copper, manganese and polonium ^84^Po_210_. Cigarette tar contains various aromatic ring compounds (especially benzo-[a] oyrene).
7	Vinyl chloride [CAS: 75-01-4]: CH_2_ = CHCl,
	It is a manufactured substance that is used to prepare polyvinyl chloride to make plastic products for use in food packaging, medical products, appliances, cars, toys, credit cards and rainwear, and leaches into air and water.

The chemicals listed are those for which strong evidence exists.

*Source*: Chandrasoma, P. and Taylor, C.T. (2000) Concise Pathology, 3rd edn. East Norwalk, CT: Appleton& Lange; Kumar, V., Abbas, A.K. and Aster, J.C. (2014) Robins and Cotran Pathologic Basis of Disease, 9th edn. Philadelphia, PA: W.B. Saunders.

Virus is an ultramicroscopic virion wrapped in protective coating of protein, infectious agent, obligate intracellular parasites whose replication depends on its core DNA or RNA and protein synthetic process of the host cell for growth in tissue culture [104]. The major pathogenic of viruses are adenoviridae, flaviviridae, hepadnaviridae, herpesviridae, homyxoviridae, papovaviridae, paramyxoviridae, picornaviridae, polyomaviridae, orthomyxoviridade, rhabdoviridae and togaviridae [105]. The viral infection commonly reaches optimal time as a function of replication as clinical symptoms appear [106] , and the replication consists of the following steps: (1) attachment to and penetration of susceptible cell, (2) disassembly of nonstructural proteins to make nucleic acid available for virus multiplication, (3) synthesis of RNA or DNA through transcription and translation (Figure 2), (4) synthesis of structural and functional proteins, and (5) assembly and release mature viral particles from the cell [81,104]. Table 2 shows antiviral chemical agents that would clinically block virus replication when they are administered on the inset of disease, i.e. chemoprophylaxis.

**Table 2:** **Antiviral agents and some of their properties**

**Drug**	**Description**	**Used for**
**Purine and Pyrimidine analogues**		
Atripla	An antiretroviral contains non-nucleoside (**efavirenz**) and two nucleosides (**emtricitabine** and **tenofovir disoproxil fumerate**) reverse transcriptase inhibitor.	A fixed-dose regimen or in combination with other antiretroviral drugs (rifampin) for the treatment of HIV infection.
Azidothymidine: Combivir	An anti-HIV drug containing nucleoside reverse transcriptase inhibitors, containing **zidovudine** and **lamivudine.**	In combination with other antiretroviral drugs for treatment of immunodeficiency for HIV infection.
Retrovir	A nucleoside reverse transcriptase.	In combination with other retroviral drugs to treat HIV infection.
Trizvir	An antiretroviral, nucleoside reverse transcriptase inhibitor.	In combination with other drugs for treatment of HIV infection.
Complera	An antiretroviral contains non-nucleoside (**rilpivirine**) and two nucleosides (**emtricitabine** and **tenofovir disoproxil fumerate**) reverse transcriptase inhibitor.	A fixed-dose regimen or in combination with other antiretroviral drugs for the treatment of HIV infection.
Cymevene	An antiviral preparation which is a DNA polymerase inhibitor, containing **ganciclovir**.	Cytomegalovirus-CMV in AIDS patients with reduced immunity.
Emtriva	A nucleoside reverse transcriptase inhibitor that contains **emtricitabine**.	In combination with other antiretroviral drugs for treatment of HIV infection. It is similar to **lamivudine** and cross-resistance between the two is near similar.
Epivir	A nucleoside reverse transcriptase inhibitor, containing **lamivudine**.	In addition to other antiretroviral drugs to control and treat HIV infection.
Harvoni	A nucleoside reverse transciptase inhibitor that contains both **ledipasvir** and **sofosbuvir**.	An antiviral drug to treat chronic Hepatitus C.
Herpid	An antiviral preparation containing **idoxuridine**.	Skin infections caused by Herpes zoster and Herpes simplex.
Hivid	A nucleoside reverse transcriptase inhibitor, containing **zalcitabine**.	A combination therapy with antiretroviral drugs for treatment of HIV infection.
Lamivudine: Combivir	A nucleoside reverse transcriptase inhibitor that contains both **lamivudine** and **zidovudine**.	In combination with other antiretroviral drugs for treatment of HIV infection.
Epivir	A nucleoside reverse transcriptase inhibitor.	In combination with other antiretroviral drugs for treatment of HIV infection and disease.
Trizivir	A nucleoside reverse transcriptase inhibitor that contains both **lamivudine** and **zidovudine**.	In combination with other antiretroviral drugs for treatment of HIV infection.
Zeffix	A nucleoside reverse transcriptase inhibitor.	Chronic Hepatitis B and evidence of viral replication in patients with liver disease, inflammation and fibrosis.
Rebetol	An antiviral preparation that contains **tribavirin**.	In combination with **interferon** (pegintron) for the treatment of chronic Hepatitis C.
Stribild	An antiretroviral tablet contains non-nucleoside (**elvitegravir**) integrase strand transfer inhibitor, mechanism-based inhibitor of cytochrome P450 (**cobicistat**) and two nucleosides (**emtricitabine** and **tenofovir disoproxil fumerate**) reverse transcriptase inhibitor.	A fixed-dose regimen for the treatment of HIV infection in adults who are antiretroviral treatment-naïve.
Videx	A nucleoside reverse transcriptase inhibitor that contains **didanosine**.	In combination with other antiretroviral drugs for treatment of HIVinfection.
Vira-A	An antiviral medication that contains **vidarabin**.	Ointment treatment caused by Herpes simplex. It is an alternative to acyclovir.
Viracept	An antiviral protease inhibitor that contains **nelfinavir**.	In addition to other antiretroviral drugs for treatment of HIV infection.
Viread	An antiviral nucleotide analogue that contains **tenofivir**.	In combination with other retroviral drugs for treating Hepatitis B and HIV infected patients with virological failure.
Virgan	An antiviral eye gel preparation that contains **ganciclovir**.	Acute inflammation of cornea, keratitis.
Viroptic	An antiviral medication that contains **trifluridine**.	Eye drops treatment caused by mild viral infection.
Zeffix	A nucleoside analogue that contains **lamivudine**.	Chronic Hepatitis B and evidence of viral replication in patients with liver disease, inflammation and fibrosis.
Zerit	A nucleoside reverse transcriptase inhibitor that contains **stavudine**.	In combination with other antiretroviral drugs for treatment of HIV infection.
Zovirax	An antiviral DNA polymerase inhibitor that contains **acyclovir**.	Treatment and suppression of infections of skin and mucous membranes caused by Herpes simplex, Herpes zoster and varicella.
Other drugs		
Abacavir : Trizivir	An antiretroviral,nucleoside reverse transcriptase inhibitor that contains **abacavir**, **lamivudine** and **zidovudine**.	In combination with other antiretroviral drugs for the treatment of HIV infection.
Ziagen	A nucleoside reverse transcriptase inhibitor.	In combination with other antiretroviral drugs for treating HIV infection.
Amanatadine hydrochloride:	A viral replication inhibitor.	Treatment and prevention of influenza A.
Lysovir	A dopaminergic, tricyclic amine preparation.	Parkinsonism.
Symmetral		
Dolutegravir	An antiretroviral, integrase strand transfer inhibitor.	Treatment of HIVinfection in conjunction with other antiretroviral drugs.
Foscavir	A DNA polymerase inhibitor that acts in two stages to suppress the replication of cytomegalovirus; it contains **foscarnet**.	Life-threatening infections of viral origin especially those of eyes in AIDS patients with cytomegalovirus retinitis. Also, infections of mucous membranes and skin caused by Herpes simplex and AIDS patients who have not responded to treatment with acyclovir.
Gamma globulin:	A preparation of human normal immunoglobulin (5%).	Immunoglobulin replacement therapy in patients who are immunodeficient, including, including various inherited disorders like hypogammaglobulinaemia (low levels of gammaglobulin in blood), agammaglobulinaemia (absence of gammaglobulin in blood). Also, thrombocytopenia purpera (bleeding disorder), and helping to prevent recurrent infection by HIV infection.
	A preparation of human normal immunoglobulin (16%).	
Flebogamma	A preparation of human normal immunoglobulin, freeze-dried powder comprising 0.5 g, 2.5 g, 5.0 g and 10.0 g in bottles with diluents for reconstitution and injection.	
Gammabulin	A preparation of human normal immunoglobulin (5%).	Antibody deficiency syndrome.
	A freeze-dried preparation of human normal immunoglobulin.	Guillain-Barre syndrome (a severe,often rapidly progressive syndrome of muscular weakness and paralysis, believed to be an autoimmune disease), Kawasaki syndrome (a disorder affecting lymph nodes), thrombocytopaenia purpera (a clotting disorder involving blood platelets).
Gammagard	A preparation of human normal immunoglobulin, available as a solution and powder	Replacement in various immunodeficiency states, including certain congenital conditions like thrombocytopaenia purpera. Also, prevention of recurring bacterial infections with HIV, Guillain-Barre syndrome, Kawasaki syndrome.
OctagamSandoglobulinVigam		Replacement treatment in primary and secondary immunoglobulin deficiencies, prevention of bacterial infections in children born with AIDS, thrombocytopenic purpura, Kawasaki disease, bone marrow transplants, Guillain-Barre syndrome.
Vigam		Primary agammaglobulinaemia, hypogammaglobulinaemia, and other secondary secondary immunodeficiency disorders in children with AIDS,thrombocytopenia purpura,Kawasaki disease,bone marrow transplants and Guillain Barre syndrome
Interferons alfa: Introna	A single-subtype recombinant preparation in powder.	Neoplastic disorders (malignant conditions).
Roferon-A	A preparation of interferon alfa-2a in a solution.	Hepatitis B and Hepatitis C (with or without **tribavirin**), neoplastic disorders.
Viraferon	A preparation of interferon alfa-2b ina solution.	Chronic Hepatitis B and chronic Hepatitis C infections.
Interferons beta: Betaferon	A preparation acting on the immune system.	Relapsing-remitting multiple sclerosis, and secondary progressive multiple sclerosis.
Rebif	An immunimodulator of interferon beta-1a.	Multiple sclerosis with relapses.
Interferons Gamma: Immukin	A preparation of recombinant human interferon gamma- Ib.	Additional treatment (with antibiotics) to lessen the incidence of serious infections acquired by patients with chronic granulomatous disease (any disese that gives rise to masses of granulation tissue, e.g.tuberculosis and leprosy).
Paclitaxel	A taxoid antineoplastic agent and mitotic inhibitor that is based on plant alkaloid taxane or 11 (15- > 1) abeotaxane.	Treatment of tumor cancers: breast, ovarian, lung, bladder, prostate, melanoma, esophageal and Kaposi sarcoma.
Rifadin	An antibiotic and antimalarial preparation that contains **rifampicin**.	Prevention of meningococcal meningitis, carriers of Haemophilus influenza, additional therapy for brucellosis, Legionnaire’s disease and serious staphylococcal infections, tuberculosis and mycobacterial infections, leprosy.
Triumeq	An antiviral medication that contains a non-nucleoside integrase strand transfer inhibitor (**dolutegravir**), and two nucleoside reverse transcriptase inhibitors (**abacavir** and **lamivudine**).	A single-pill regimen for treatment HIV infection.
Viramune	A non-nucleoside reverse transcriptase inhibitor that contains **nevirapine**.	In combination with other antiretroviral drugs for treatment HIV infection, progressive and/or advanced immunodeficiency.

Both DNA and RNA viruses can cause neoplasia. DNA viruses insert their nucleic acid directly into the genome of host cell and the virus replication normally ensues. Examples: Papilloma, Herpes simplex, Molluscum contagiosum,and Hepatitis B. RNA viruses require RNA-directed DNA polymerase (reverse transcriptase), an enzyme that causes production of a DNA copy of the RNA viral genome. Retroviruses examples: human T lymphocyte virus type I- HTLV-I, acquired immune deficiency syndrome –AIDS, human immunodeficiency virus –HIV.

*Adopted from*: Medicines & Prescription Drugs [102] New Lanark,ML: Geddes & Grosset; Pisano, D.J. and Mantus, D.S. (2008) FDA Regulatory Affairs: A Guide for Prescription Drugs, Medical Devices and Biologics, 2nd edn. New York, NY: Informa Healthcare USA, Inc.; Katzung, B.G., Masters, S.B. and Trevor, A.J. (2011) Basic & Clinical Phamacology, 12th edn. New York, NY: McGraw-Hill Medical Division.

### Methodology

On infection, a viral RNA permeates human host cell membranes, whereupon it is either destroyed by several cellular RNases [E.C.3.1.26.4 RNase H; E.C. 3.1.26.5 RNase P; E.C. 3.1.27.3 RNase T_1_; E.C. 3.1.27.1 RNase T_2_; E.C. 3.1.27.4 RNase V_2_; E.C.3.1.27.8. RNase V_1_ and others], or it binds to ribosomes and it grows and divides to make proteins at an unregulated quickened pace [107,108]. The process of transcription cycle that consists of: preinitiation, initiation,promoter clearance, elongation and termination have a significant impact on the growth potential of tumours [109]. The failure of the host cell to recognize and destroy the viral infection is caused by the lack of particular co-stimulated molecules that aid in the way antigens react with lymphocytes [110-112]. Hence, the basic research into cancer entails identification of causes and developing strategies for prevention, diagnosis, treatments and cure [113-116]. The research spans chemotherapy, hormone therapy, immune therapy, nanomaterials, palliative surgery, radiation therapy and combined treatment modalities [77,94,117-121]; and the methods of evaluation were mainly: (1) cytological methods (exfoliative and aspirational cytology), (2) flow cytometry, (3) hystological methods, (4) immunohistochemistry, (5) molecular diagnosis (aptamers), (6) tumor markers (hormones (calcitonin, catecholamine & metabolites, ectopic, human chronic gonadotropin-HCG), oncofetal antigens (α-fetal protein, carcino embryonic antigen), iso enzymes, specific proteins, mucins and glycoproteins, new molecular markers).

RNA phage of each step of protein synthesis that could be possible to control, is indicative of individual inter-related biosynthesis of a given protein [122]. The rate of initiation complex formation dictates the amount of each of the viral proteins [98,123]. The RNA polymerases begin to replicate the viral RNA, a process of the most central mediators of malignant transformation [32,124]. RNA polymerase I [125], RNA polymerase II [50,126,127] and RNA polymerase III [128,129] transcribe protein-coding genes and they interact with factors involved in the synthesis of precursor rRNA 45S, chromatin remodeling, transcriptional activation, elongation, and RNA processing.

The multicellular eukaryotic human enzymes can be purified using isolated organelle, like nucleus, nucleoli, mitochondria and other internal organelles as a starting material, though simultaneous recovery of all three RNA polymerases is not always feasible due to diffusible nature of some of nuclear enzymes [18,110]. Jacob and Rose [130] had extensively reviewed the methods of solubilization, purification and difficulties of mammalian RNA polymerases.

HeLa cells were often the source for RNA polymerase complexes [131]; mitotic cells and cancer tissues with corresponding normal tissues that can be collected and frozen in liquid nitrogen at −80°C, are viably preserved until essayed [127,132]. Histopathological examination is done on the 10% formalin-fixed, paraffin-embedded tissue specimens which is an invaluable resource for clinical research though the nucleic acids extracted are fragmented and chemically modified making them challenging to use in molecular studies [133-135]. Histopathological observations are utilized in progression, metastatic susceptibility, therapeutic and radiation therapy sensitivity and prognosis [136,137]. Interestingly, the Raman spectroscopy non-invasive technique enables the observation of intracellular biological molecules without fixation or labeling procedures in situ [137,138].

The bicinchoninic acid kit protein quantification assay [139] that is widely used to determine protein concentrations in the region 25-2000 μg/ml. The cells are suspended and lysed in hypotonic buffer (20 mM Tris–HCl [pH 7.5], 1 mM CaCl_2_, 1 mM MgCl_2_, 1 mM ZnCl_2_ containing 1% Triton X-100) for 5 min on ice; they were then separated into nucleolar and nucleoplasmic fractions by zonal centrifugation in sucrose [140] at 15,000 rpm for 15 min at 4°C, and the supernatant was collected and frozen at −20°C until re-use [50,132]. This method is a modified form of the Lowry et al. [141] and Bradford [142] methods that are also widely used dye-binding chromogenic protein variations assays [143]. The Bradford [142] protein assay is based on the association of specific amino acid residues, arginine, lysine, and histidine, with non-conjugated groups of Coomassie brilliant blue G-250 dye, in an acidic environment. When the dye-protein complex is formed, the pK_a_ of the red-brown acidic solution converted to blue and it measured at 595 nm. Bradford dye is convenient protocol to use, fast and relatively sensitive, but several compounds can interfere with the assay against typical standard curves for bovine serum albumin and bovine gamma globulin [144]. The other Lowry et al. [141] protein assay lies in the formation of peptide nitrogen(s) complex with Cu^2+^ under alkaline conditions (pH 10.0-10.5), and the subsequent reduction of the Folin-Ciocalteay phosphomolybdic phosphotungstic acid reagent to heteropolymolybdenum blue at spectra 750 nm, though ethylenediaminetetraacetic acid (EDTA) can interfere with chromophore production [145].

Radio-immunohistochemistry is a very sensitive in *vitro* technique in which a traceable radioactive isotope tags a marker to detect, identify and quantitate the concentration of specific ochemical neoplasia substance(s) [134,146-149]. Radioimmunoassay has been developed to identify and quantitate the concentration of RNA polymerase I [150], RNA polymerase II [151], RNA polymerase III [152] and mRNA [153]. Although it is less sensitive than enzymic activity as a measure of reverse transcriptase, but it can detect antigen using minute amounts of protein and in the presence of inhibitors for an RNA tumor virus-producing cells [154].

The three RNA polymerases transcribe the genome in the cell nuclei. The RNA polymerase II that is responsible for synthesizing mRNA and a large variety of noncoding RNAs, is mostly important; RNA production in growing cells is carried out by RNA polymerase I that transcribes the precursor of large rRNA, and by RNA polymerase III that transcribes rRNA, tRNA, and some noncoding RNAs [155]. Hossenlopp et al. [156] have used anti-polymerase I serum to classify the three RNA polymerases in order of their inhibition: I > III > I, indicating that polymerases I and II are more closely related than polymerases I and II [32,157].

### Findings and interpretations

The differential effect of selective inhibition on nuclear and nucleoplasmic RNA sysnthesis is related to the existence of distinct nuclear and chromosomal RNA polymerases that cause mitotic-like biochemical and morphological responses. Also. ribosome synthesis in HeLa cells is shown to be controlled at the level of processing rather than at the level of 45S RNA transcription where chemical agents would cause the physiological and structural transitions of viral mitosis [158,159]. Table 2 lists some of these therapeutic agents that can be considered to interfere with the process of virus replication.

In conclusion, the advent of sequencing human genome has facilitated the impressive advances in diagnosis, prognosis and treatment methodologies of invasive human tumor cells. A new area of research into chemical agents that interfere with mitosis-related cell death (apoptosis), are able to denature chemotherapy resistant neoplastic cells and inhibit protein expressions. Giri and Kumar [55,160] have reported that the over expression of neo-poly (A) polymerase in human cancer cells signifies the importance of polyadenylation in cancer cellular events. The specificity of an electrostatic interaction between RNA and natural alkaloids or their synthetic analogs is found to be capable of inducing self-structure in polyadenelation. Hence, new novel compounds that exhibit excellent binding affinity to many RNA structures, can be utilized to modulate poly (A) structure in developing RNA targeted cancer therapeutics [161,162]. The nanoparticles of aptamers are also emerging to target the reaction of specific antigen epitopes with their binding sites. These are promising techniques in clinical diagnosis and therapy. Such new insights into genetics of tumors have prompted ground-breaking insights into development of new drugs that can treat, shrink and coax given tumors into prolonged remission.
